# 

**Published:** 1883-04

**Authors:** 


					﻿Vol. IV.
APRIL, 1883.
No. 4.
THE
ntotiidtnlPraelitionei
A Monthly Journal,
DEVOTED TO
MEDICINE, SURGERY, OBSTETRICS, DENTISTRY, PATHOLOGY
AND POPULAR SCIENCE.
EDITORS :
LEIGH IT. TTTTISrT, B.Sc., JVE.TD.
■W. CL BARRETT, TD.ID.S., M.D.
New York:
THE COLUMBIA PUBLISHING CO.,
42 DUANE STREET.
$2.50 Per Annum in Advance. -	- Single Copies, 25 Cents.
Entered at the Post-Office, New York City, as Second-Class matter.
				

